# Supplementary material for: Comparative Computational Modeling of the Bat and Human Immune Response to Viral Infection with the Comparative Biology Immune Agent Based Model
Source: Viruses. 2021 Aug 16;13(8):1620. doi: 10.3390/v13081620 (PMC8402910; doi:10.3390/v13081620)
Supplement: Supplementary file 1 [file viruses-13-01620-s001.zip › viruses-1299052-si final.pdf]

Supplementary Material Text S1: Introduction to running the CBIABM

We are providing this documentation along with the ability to download the entire CBIABM NetLogo model. We encourage interested parties to interact with the model, and even modify or repurpose portions of its code if the wish. However, we would request that for any of the CBIABM that is repurposed into new models that appropriate attribution be provided.

The CBIABM is implemented in NetLogo, which can be downloaded at https://ccl.northwestern.edu/netlogo/download.shtml. NetLogo is a self-contained agent-based modeling environment and is very well documented with an excellent tutorial. Interested readers are strongly encouraged to look through the User Guide https://ccl.northwestern.edu/netlogo/docs/ prior to opening and running the CBIABM. This Appendix is not intended to replace the Tutorial, and will assume some familiarity with NetLogo terminology.

After the NetLogo package has been installed and the CBIABM file downloaded from http://www.github.com/An-Cockrell/Comparative-Biology-Immune-ABM (making sure that the CBIABM file has the appropriate .nlogo suffix), the model can be opened by double-clicking the CBIABM file. The model should look like this:

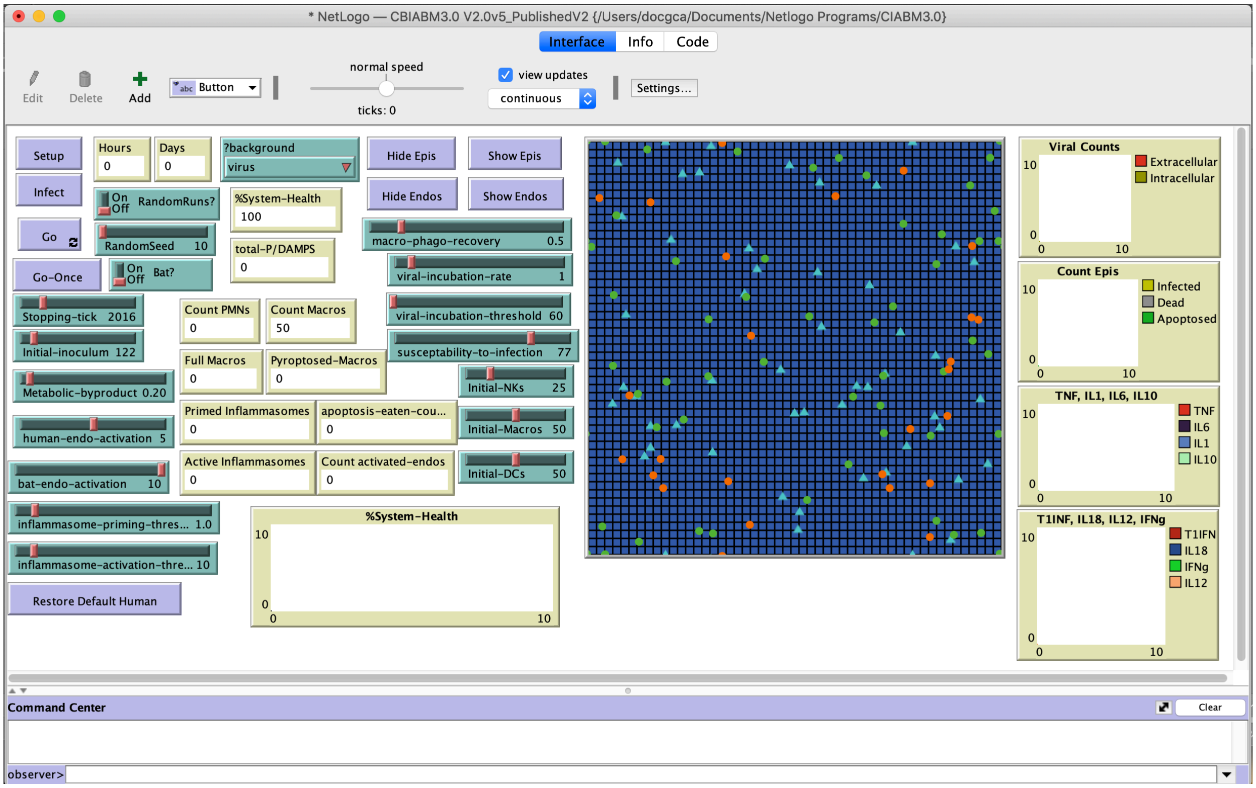

This is the User Interface where the user interacts with the CBIABM to run the model and observe its behavior. There are several widgets on the Interface:

1. Buttons (Light Blue). These are used to call specific functions when pushed, either one time or repeatedly ("Forever" buttons denoted by the small circular arrows in the right lower corner of the button; these run the function until the button is pushed again to stop it).
2. Parameter Controls (Green). These are either Sliders (values across a range), Choosers (specific arguments, usually strings/words/names) and Switches (On/Off).
3. Outputs (Tan bordered): These are either Monitors (report specific values) or Plots (time series graphs). Other plotting options are available in NetLogo but not employed in the CBIABM.

You will note that the Sliders, Choosers and Switches have values present with the model opens: these are default values for the base Human Parameterization. These values can be reset to default by pressing the "Restore Default Human" button in the left lower corner of the interface (exception to this is the "Initial-Inoculum" value, which needs to be manually reset).

The widgets are grouped for ease of use. The following Figures highlight specific groupings and their general roles.

Run Controls:

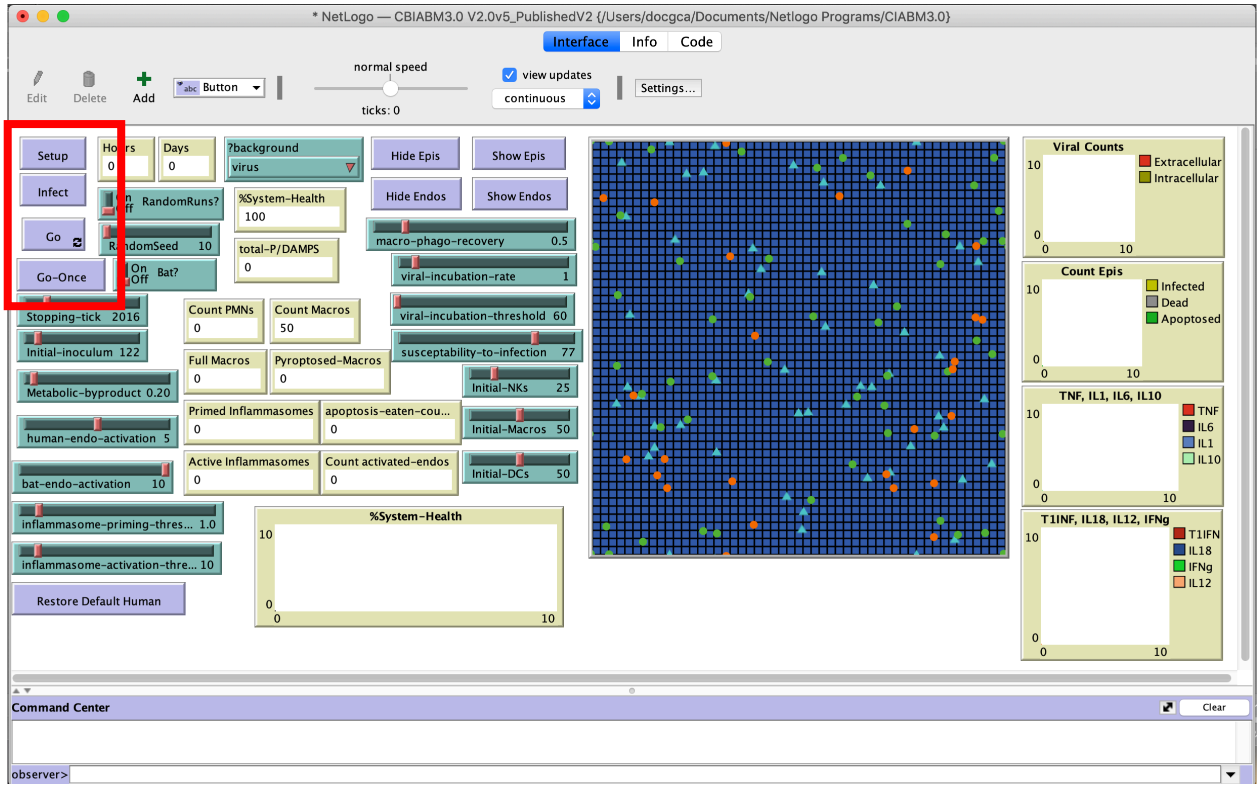

The red box above outlines the main user buttons used to run the CBIABM. "Setup" initializes the model, "Infect" applies the initial viral load (set by the "Initial Inoculum" slider) and "Go" runs the model until the either the button is pushed again, or the ticks/steps reach the value set by the "Stopping-tick" slider (default = 2016 ticks or 14 days). The sequence for running a single run of the model involves pushing the "Setup" button, which resets the model, then

“Infect” button, which administers the perturbation, and then “Go” button. The “Go” button is a “forever” button that will run until pushed again to stop it. The “Go-Once” button runs a single step. The speed at which the model runs can be adjusted with the slider in the grey toolbar above the visual interface labeled “normal speed.” This allows the user to slow the simulation down to look at particular dynamics or speed the simulation up to save computing time.

The World view, consisting of the 51 x 51 square grid, can be seen on the right side of the User Interface. The following is a key to the agent shapes seen on that view:

Blue Squares = Healthy Epithelial Cells
Yellow Squares = Infected Epithelial Cells
Grey Squares = Epithelial Cells killed by necrosis
Grey Pentagons = Epithelial Cells killed by apoptosis
Green Circles = Macrophages
Large Green Circles = Macrophages at phagocytosis limit
Orange Circles = NK Cells
Light Blue Triangles = Dendritic Cells
Pink Square Outlines = Activated Endothelial Cells
Small White Circles = PMNs

At initialization the entire world-space is filled with Healthy Epithelial Cells (Blue Squares). When dead Epithelial Cells of both types are cleared by phagocytosis what remains is black space, which can then be refilled be new Epithelial Cells as they heal (see main text for rules). The levels of extracellular virus and different mediators can be seen in the background behind the agents; see “Visualization Controls” below how to hide and reshow Epithelial and Endothelial Cells.

Overview Controls

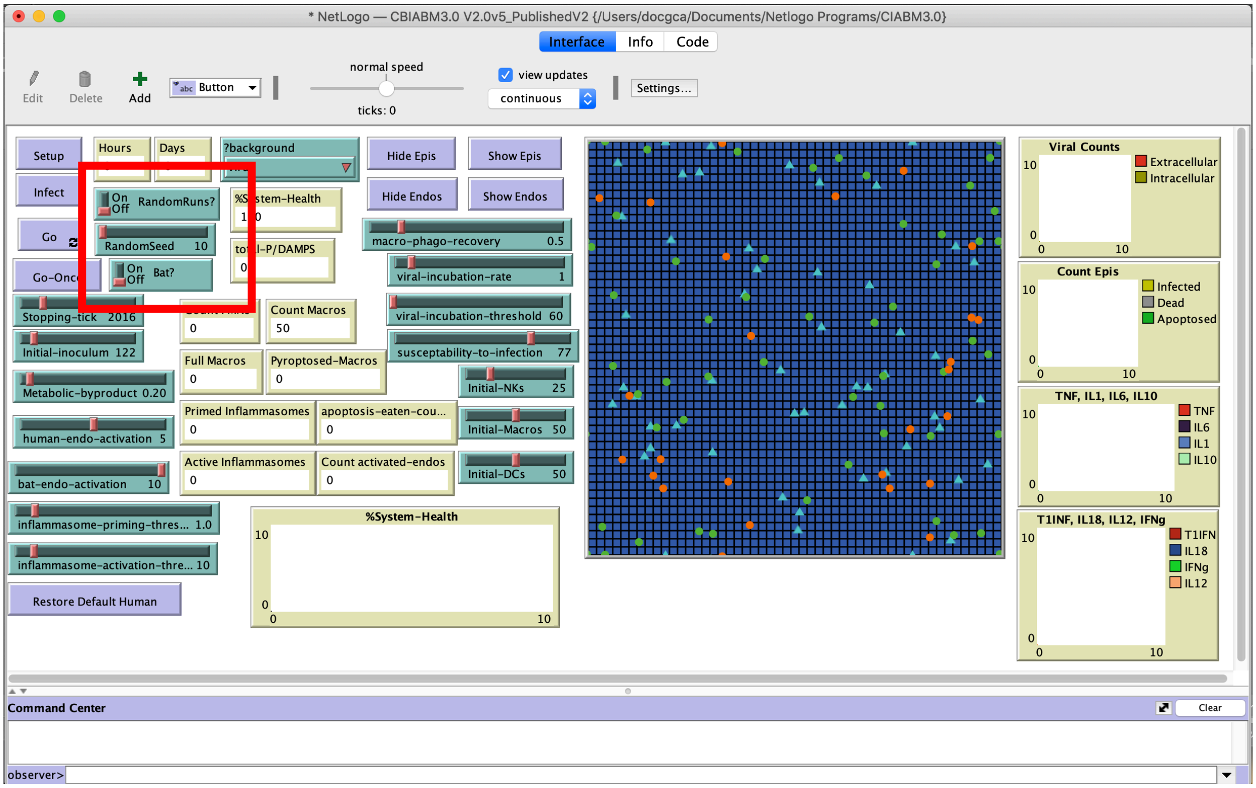

The controls in outlined in this next figure can be considered overview controls. The “RandomRuns?” switch determines whether each push of “Setup” uses a new, randomly selected random number generator seed, or uses the random number seed manually chosen with the “RandomSeed” slider immediately below. If “RandomRuns?” is set to “Off” then every time the model is reinitialized by pushing “Setup” the model will have exactly the same configuration and run exactly the same each time for a given set of parameters. This is implemented so that simulation experiments can sweep over values of “RandomSeed” to generate stochastic replicates, but with the ability to keep track of those runs so that particular ones of interest can be directly reproduced. Conversely, running stochastic replicates with the “RandomRuns?” set to “On” will create a population of runs, but no way to recreate a specific run within that group. “RandomRuns?” is also set to “Off” during development, in order to help determine if differences in model behavior are due to code changes as opposed to stochasticity in the model.

The next overview control in this figure is the “Bat?” switch, which allows the user to select whether the CBIABM is running in either Bat or Human model. Of note, this switch controls the differences in T1IFN implementation between Bat and Human, but DOES NOT change values related to the differences in metabolic rate or inflammasome priming/activation between the two species. These require modification of the following sliders: “Metabolic-byproduct” (Human = 0.2, Bat = 2.0), “inflammasome-priming-threshold” (Human = 1.0, Bat = 10) and “inflammasome-activation-threshold” (Human = 10, Bat = 50).

Parameter Controls

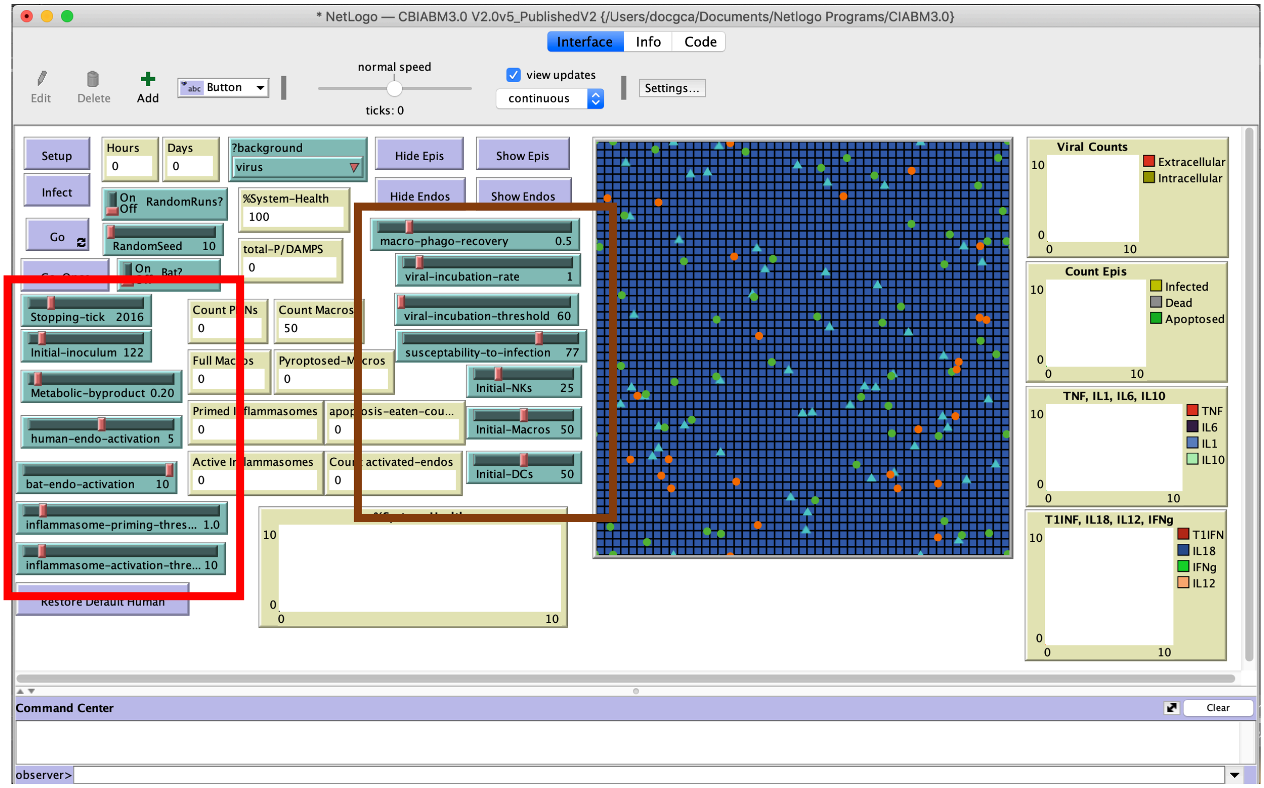

These sliders set parameter values for individual runs. Of note, the ones in the Red Box are those that are modified during the simulation experiments presented in the paper, whereas the ones in the Brown Box are values arrived at during development/calibration. The ones in the Brown Box SHOULD NOT BE MODIFIED without recognizing that they may change the established base calibration dynamics of the CBIABM. These values are to a great degree arbitrarily established early on in development and form a reference point from which subsequent rules are implemented and tuned. This emphasizes both the meaninglessness of the actual numerical values for parameters and outputs (as the CBIABM is not intended to be spatially accurate and this affects all the above factors) and the importance of the relative relationship between the parameter values in terms of model behavior. The values of the slider can be modified by either using the mouse to drag the slider to particular value or double-clicking on the slider to bring up the interface box and a specific value inputted.

These sliders can also be set using NetLogo's Behavior Space tool; users are encouraged to examine the NetLogo Programming Guide for details. However, we suggest that if simulation experiments are to be performed using Behavior Space that the specific values of the sliders be explicitly set in Behavior Space (as opposed to relying on manually setting them using the widget); this is because the widget values can be unintentionally changed when navigating over the screen. Also, setting them explicitly in Behavior Space will put this information in the output file (.csv format) produced by Behavior Space.

Visualization Controls

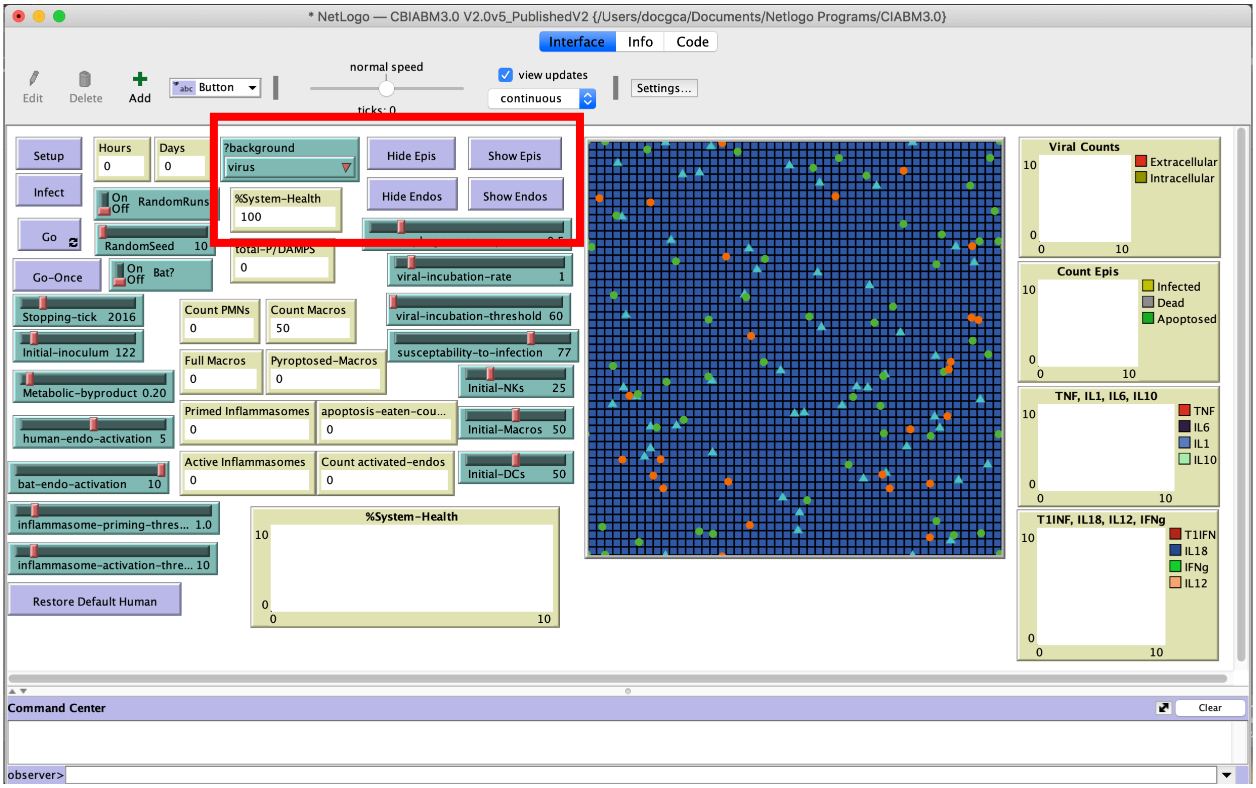

The controls in the red box in this next figure allow the user to change the visualization of the CBIABM as it runs. One of the primary benefits of NetLogo is the model visualization window in the User Interface that allows the user to see how the model is behaving. This, along with the monitors and plots, allows the user to rapidly iterate development if the model exhibits grossly un-intended behaviors, and confirm that selected functions are operating correctly via ongoing component testing. The various agents and patch variables are color coded to aid in visualization. A key is presented in the Info Tab of the model, but some specific cell/agent types are noted here. Healthy Epithelial cells are seen as blue squares, and at initialization there is one per grid space; when they become infected they turn yellow. Because the epithelial cells cover the vast majority of the background they obscure any mediators (and extracellular viral counts) present as patch variables. The distribution of these patch variables can be better seen by pushing the “Hide Epis” Button, at which point they become transparent. “Show Epis” reverses this process. Similarly, activated endothelial cells are represented by pink-outlined squares; they appear when activated but also obscure patch variables and other cell/agents is present. They can be hidden with the “Hide Endos” button and revisualized with “Show Endos.”

Performing Simulation Experiments and Reproducing Published Runs

The following information relates to the specific starting conditions and parameter sweeps for the Results in this paper. *NOTE: Simulation experiments are run with the "Random-Runs" switch "Off", stochastic replicates achieved by incrementing the random number seed by 1 to

the number of stochastic replicates desired. This is done to allow tracing of specific runs (by seed); this allows potential deeper examination of specific run that might be of interest.

For Parameter sweeps:
Baseline Bat
Bat? = true
Metabolic-byproduct = 2.0
Inflammasome-priming-threshold = 5.0
Inflammasome-activation-threshold = 50
Bat-endo-activation = 10

Baseline Human
Bat? = false
Metabolic-byproduct = 0.2
Inflammasome-priming-threshold = 1.0
Inflammasome-activation-threshold = 10
Human-endo-activation = 5

Initial Injury Sweeps (Figure 3a-b):
Initial-inoculum varies from 25-150 increments of 25, run for 14 days (2016 steps)
N = 1000 stochastic replicates
Main Comparison is distribution of %System-health across stochastic replicates at the end of the run for each Initial-inoculum

Evaluation of Effect of Endothelial Inflammasome (Figure 4)
Uses Human parameterization EXCEPT sweep from Human-endo-activation from 5 to 10 (bat level).
N = 250 Stochastic Replicates all run with Initial-Inoculum = 150, run for 14 days.
Main Comparison is distribution of %System-health across stochastic replicates with increasing Human-endo-activation.

Evaluation of Effect of Metabolic Stress on Bat Disease (Figure 5)
Uses Bat Parameterization EXCEPT sweep of Metabolic-Byproduct from 2 to 10
N = 250 Stochastic Replicates all run with Initial-Inoculum = 150, run for 14 days
Main Comparison is distribution of %System-health across stochastic replicates with increasing Metabolic-Byproduct.

As noted above, NetLogo has a built-in tool called Behavior Space. Information about Behavior Space can be found in the NetLogo User Guide; it is listed under "Features" in the Index on the left of the screen. Behavior Space can be accessed through the NetLogo interface in the drop-down menu under "Tools." Selecting Behavior Space will bring up a list of the simulation experimental conditions by specific experiment. Refer to the NetLogo tutorial to setup and run Behavior Space simulation experiments.
